# First exposure to rituximab is associated to high rate of anti-drug antibodies in systemic lupus erythematosus but not in ANCA-associated vasculitis

**DOI:** 10.1186/s13075-021-02589-6

**Published:** 2021-08-13

**Authors:** Francesca Faustini, Nicky Dunn, Nastya Kharlamova, Malin Ryner, Annette Bruchfeld, Vivianne Malmström, Anna Fogdell-Hahn, Iva Gunnarsson

**Affiliations:** 1 Department of Medicine Solna, Division of Rheumatology, Karolinska Institutet, Stockholm, Sweden; 2 Unit of Rheumatology, Karolinska University Hospital, Stockholm, Sweden; 3 Department of Clinical Neuroscience, Karolinska Institutet, Stockholm, Sweden; 4 Center for Molecular Medicine, Stockholm, Sweden; 5 Department of Health, Medicine and Caring Sciences, Linköping University, Linköping, Sweden; 6 University Hospital and Department of Renal Medicine, Karolinska University Hospital and CLINTEC Karolinska Institutet, Stockholm, Sweden

**Keywords:** Rituximab, Anti-drug antibodies (ADA), Systemic lupus erythematosus, ANCA-associated vasculitis, Disease activity, B cell counts, ADA-screening

## Abstract

**Background:**

Anti-drug antibodies (ADAs) can impact on the efficacy and safety of biologicals, today used to treat several chronic inflammatory conditions. Specific patient groups may be more prone to develop ADAs. Rituximab is routinely used for ANCA-associated vasculitis (AAV) and as off-label therapy for systemic lupus erythematosus (SLE), but data on occurrence and predisposing factors to ADAs in these diseases is limited.

**Objectives:**

To elucidate the rate of occurrence, and risk factors for ADAs against rituximab in SLE and AAV.

**Methods:**

ADAs were detected using a bridging electrochemiluminescent (ECL) immunoassay in sera from rituximab-naïve (AAV; *n* = 41 and SLE; *n* = 62) and rituximab-treated (AAV; *n* = 22 and SLE; *n* = 66) patients. Clinical data was retrieved from medical records. Disease activity was estimated by the SLE Disease Activity Index-2000 (SLEDAI-2 K) and the Birmingham Vasculitis Activity Score (BVAS).

**Results:**

After first rituximab cycle, no AAV patients were ADA-positive compared to 37.8% of the SLE patients. Samples were obtained at a median (IQR) time of 5.5 (3.7–7.0) months (AAV), and 6.0 (5.0–7.0) months (SLE). ADA-positive SLE individuals were younger (34.0 (25.9–40.8) vs 44.3 (32.7–56.3) years, *p* = 0.002) and with more active disease (SLEDAI-2 K 14.0 (10.0–18.5) vs. 8.0 (6.0–14), *p* = 0.0017) and shorter disease duration (4.14 (1.18–10.08) vs 9.19 (5.71–16.93), *p* = 0.0097) compared to ADA-negative SLE. ADAs primarily occurred in nephritis patients, were associated with anti-dsDNA positivity but were not influenced by concomitant use of corticosteroids, cyclophosphamide or previous treatments.

Despite overall reduction of SLEDAI-2 K (12.0 (7.0–16) to 4.0 (2.0–6.7), *p* < 0.0001), ADA-positive individuals still had higher SLEDAI-2 K (6.0 (4.0–9.0) vs 4.0 (2.0–6.0), *p* = 0.004) and their B cell count at 6 months follow-up was higher (CD19 + % 4.0 (0.5–10.0) vs 0.5 (0.4–1.0), *p* = 0.002). At retreatment, two ADA-positive SLE patients developed serum sickness (16.7%), and three had infusion reactions (25%) in contrast with one (5.2%) serum sickness in the ADA-negative group.

**Conclusions:**

In contrast to AAV, ADAs were highly prevalent among rituximab-treated SLE patients already after the first course of treatment and were found to effect on both clinical and immunological responses. The high frequency in SLE may warrant implementations of ADA screening before retreatment and survey of immediate and late-onset infusion reactions.

## Introduction

Since the introduction of the chimeric monoclonal anti-CD20 antibody rituximab (RTX) in the management of haematological malignancies [1], its use has been extended to several immune-mediated diseases including rheumatoid arthritis (RA) [2], anti-neutrophil cytoplasmic antibody (ANCA)-associated vasculitis (AAV) [3], systemic lupus erythematosus (SLE) [4] and multiple sclerosis (MS) [5]. In SLE, the drug is used as off label therapeutic, after two large randomized clinical trials having failed to meet their primary endpoints [6, 7]. Nevertheless, there is a substantial body of literature that supports its use in SLE, especially as add-on medication in patients with refractory organ manifestations, mainly in lupus nephritis [8, 9].

The development of anti-drug antibodies (ADAs) is the result of an unwanted immune response which can occur against all biologicals including RTX and can impair the efficacy and safety of the treatment. The issues related to the potential immunogenicity of the biologics were recognized early in the process of their development. As a result, several efforts have been performed at a preclinical level to reduce the immunogenic potential of the different molecules. The primary reason for the biologics to be immunogenic resides in the proteinaceous nature of these molecules, their structure and the post-translational changes acquired in the production process [10].

RTX, as a chimeric antibody, can potentially carry in its mouse-derived variable regions several immunogenic epitopes that may elicit immune response in the individuals receiving the drug [11]. As a consequence, ADAs can recognize the target-binding domain of the molecule (i.e. recognize the idiotype), inhibiting the action of RTX. In addition, ADAs can also bind to other epitopes in the molecular structure of the drug, and through the formation of immune complexes, they can alter the pharmacokinetics through accelerated drug clearance, which in turn can reduce the clinical efficacy of the drug [10] or lead to infusion reactions.

In addition to the type of monoclonal antibody, the rate of occurrence of ADAs can also depend on other patient- or drug-related factors, such as the route and frequency of administration of the drug, the different dosing regimens, the presence of co-medications and the underlying disease. Indeed, some systemic inflammatory diseases appear more prone to the development of ADAs than other illnesses [12].

In this respect, there is data which suggests that in RA, the occurrence of ADAs to RTX is affecting 4–11% of treated patients [2]. In contrast, in patients with relapsing–remitting MS, the proportion of patients developing ADAs to RTX has been shown to be as high as 37% and high titres of ADAs were associated with less profound B cell depletion or earlier B cell repopulation [13].

In SLE, data concerning the rate of development of ADAs against the drug is still limited. From the clinical trials that have been carried out, SLE appears to be associated with both high rates of ADAs and high titres [4, 6, 7] but their clinical relevance remains unclear. Longitudinal studies suggest that despite an initial response to RTX, ADA development may be a significant determinant of a secondary loss of efficacy at retreatment [14]. Alternatively, ADAs may be predictive of the occurrence of infusion reactions at retreatment, as shown by a recent observational study [15]. Studies addressing both the prevalence and impact of ADAs to RTX in SLE are still few and further research is needed.

Similarly, there is limited data available clarifying the impact of RTX immunogenicity in the context of AAV. To date, immunogenicity has only been evaluated in a small prospective study enrolling eleven AAV patients [16] and in a randomized clinical trial (RCT) in which 23% of the patients were found positive for ADAs within 18 months from treatment initiation, although the clinical implications of immunogenicity were unclear [3].

Further studies in a real-world setting are needed to confirm these findings and to better understand the clinical implications of ADA development, their impact on the safety profile of RTX and on its efficacy in systemic diseases such as SLE and AAV. Therefore, the aims of the present study were to elucidate the frequency of ADAs against RTX in two cohorts, one of SLE and one of AAV patients and, if ADA impact the intended pharmacodynamic effect of RTX in reducing the B cell count.

Secondary aims of this study were to identify risk factors for ADA development in these cohorts and to evaluate the association of ADAs with adverse events at retreatment in a clinical setting. Finally, we wanted to explore, if the development of ADAs was associated with subsequent lack of response on retreatment.

## Methods

### Patients

#### SLE patients

SLE patients included in the study belong to a clinical cohort prospectively enrolled at the Rheumatology Clinic at Karolinska University Hospital, Stockholm. All patients fulfilled the 1982 ACR criteria for SLE [17], were newly initiated on RTX and had a pre-treatment (baseline) sample, and at least one post treatment sample available (preferably at 6 months after first infusion), obtained at RTX trough prior to any subsequent infusions. A total of 66 SLE patients met the inclusion criteria and were included in the study.

#### AAV patients

AAV patients diagnosed and classified according to the EMA (European Medicines Agency) algorithm [18] were recruited from a larger cohort study which has been previously described [19].

In this cohort, patients were evaluated, and blood samples were collected at inclusion and at a scheduled 6 months follow-up visit. We retrospectively identified those, among the included patients who had received RTX, either at inclusion or within the 6 months follow-up of the cohort study design, and who had available samples taken before (baseline) and after treatment initiation. Since only 22 AAV patients had been treated with RTX, we also included additional samples from 19 AAV patients unexposed to RTX.

All patients provided informed consent prior to enrolment in the cohorts. This study was approved by the Stockholm ethics committee and conducted in accordance with the Helsinki declaration.

#### Clinical data

Clinical and demographic data were retrieved from the electronic clinical charts. For all patients, data on age, gender, disease duration, main indication for RTX administration, dose and treatment schedule and associated treatments was collected. For SLE patients, concomitant treatment at RTX initiation and previous treatments including corticosteroids (CS) and anti-rheumatic disease-modifying drugs (DMARDs) were included in the data collection. Disease activity was measured using the SLE Disease Activity Index-2000 (SLEDAI-2 K) [20]. For AAV patients, the Birmingham Vasculitis Activity Score (BVAS) was used [21].

### Rituximab administration

In both cohorts, original RTX (Mabthera ®) was administered primarily using one of two different regimens including the haematological approved dosage of 375 mg/m^2^ of body surface area (BSA) every week over 4 weeks in accordance to previously published studies for both SLE [22] and AAV [3], or the RA approved dosage of 1 g 2 weeks apart. In select cases, lower doses of 500 mg 2 weeks apart were also used. Also, patients could receive concomitant intravenous cyclophosphamide, either as two infusions of 750 mg or according to Euro-Lupus [23] or NIH (National Institutes of Health) protocol [24]. The addition of 6-metylprednisolone pulse therapy of 500–1000 mg daily over 3 days was decided by the treating physician according to clinical needs.

### Laboratory investigations

Baseline and 6 months follow-up laboratory measurements including total leucocytes, total lymphocytes and lymphocytes subpopulations of CD19 + cells were retrieved from clinical records where available. These measurements were conducted according to clinical routine. B cell depletion was defined as a total CD19 + positive cell count of < 0.01 cells/µL and a percentage of CD19 + positive cells < 0.5 out of total lymphocytes according to laboratory routine.

Additional laboratory measurements included in the dataset were complement levels (C3 and C4 fractions) and serum anti-dsDNA titres. These were performed using different methods over the years, thus with different normal ranges. Therefore, these results were recorded as a categorical variable in the dataset (complement normal/low, anti-dsDNA positive/negative).

All laboratory analyses were performed at the Clinical Chemistry and Clinical Immunology Laboratory at the Karolinska University Hospital.

### Evaluation of renal response

For SLE patients treated for lupus nephritis as the main indication and subsequently retreated, data on proteinuria (assessed as 24 h proteinuria (grams/24 h) or as urine-albumin to creatinine ratio, U-ACR, expressed in mg/mmol) and creatinine levels (mmol/L) were collected in order to assess renal response after retreatment (6 and 12 months, respectively), according to commonly used definitions [25].

### Adverse events

Clinical charts were reviewed for the occurrence of adverse events including infusion reactions (IR) at time of RTX retreatment. IR were defined according to their severity and modality to be handled in accordance with the Common Terminology Criteria for Adverse Events (CTCAE, v. 5).

Serum sickness was defined as a late-onset type III hypersensitivity reaction, characterized by the occurrence of fever, cutaneous rash and/or arthralgia/arthritis which usually occurs within 5–10 days after exposure to RTX [26, 27].

### Detection of ADA

The presence of ADA against RTX was determined with an in-house validated bridging electrochemiluminescent (ECL) immunoassay, using the Meso Scale Discovery® (MSD) platform as previously described [13].

RTX-naïve samples (SLE; *N* = 62, four samples excluded due to insufficient volume of sera available; and AAV; *N* = 41) were used to establish SLE and AAV specific screening, confirmatory and titration cut-points for the immunoassay and to screen for naturally occurring ADA at baseline.

Follow-up samples (SLE; *N* = 66, AAV; *N* = 22) were used to evaluate for ADA development after RTX exposure. In brief, ADAs were detected through a three-tiered testing approach. Samples detected as positive for ADAs in the screening assay, using the disease-specific assay cut points, were subsequently assessed in a competitive assay to confirm the specificity of ADAs against RTX. The third step included titration of confirmed positive samples to determine ADA levels. The final titre was expressed as arbitrary units per millilitre (AU/mL). Confirmed positive samples below the titration cut point were given a titre of < 2 AU/mL.

### Data analysis

The primary outcome measures were ADA status (positive/negative) and titre (AU/mL) of a patient follow-up sample as determined using the bridging ECL assay.

A cut-off for lower ADA titres (lower or equal to 4 AU/mL) and higher ADA titres (above 5 AU/mL) was adopted in accordance with previous studies [13].

In brief, in descriptive analysis, categorical variables are presented as numbers and percentages, and continuous variables are described as median and interquartile range (IQR). A univariate analysis was conducted to ascertain the relationship between ADA development and baseline characteristics. Similarly, 6-month follow-up measurements of CD19 + cells (expressed as percentage out of total lymphocytes or absolute counts (*N* × 10^9^9^/L) in the peripheral blood) were compared when available with respect to the primary outcome.

To investigate if the presence of ADA was associated with adverse events or infusion reactions, clinical data from SLE patients who were retreated with RTX were evaluated and compared with ADA status and category.

Correlations between continuous variables were explored through Spearman’s test. For the analysis of the association between categorical variables, the Fisher’s or chi-square test were used as appropriate. To compare differences between groups, central measures of continuous variables were compared using the Mann–Whitney test as appropriate. To compare between time points within groups, Wilcoxon signed-rank test was applied.

Statistical analysis was conducted using SPSS software version 27. To produce graphs and figures some statistical tests were repeated using GraphPad Prism version 8. *P* values < 0.05 were deemed as significant.

## Results

### Clinical characteristics of the enrolled patients

Patient demographics and clinical characteristics for both diseases are summarized in Table 1, and additional clinical information on SLE patients is provided in Table 2.

**Table 1:** Baseline characteristics of the RTX-treated SLE and AAV patients

	**SLE (*n* = 66)**	**AAV (*n* = 22)**
**Clinical features**		
Females (*n*, (%))	60 (90.9)	12 (54.5)
Age (median (IQR))	36.3 (29.8–50.3)	62.0 (44.0–70.5)
Disease duration (median (IQR))	7.9 (2.6–14.3)	1.5 (0–6.5)
**Disease activity**		
SLEDAI-2K^a^ (*n* = 65) (median (IQR))	12.0 (7.0–16.0)	–
BVAS (*n* = 17) (median (IQR))	–	15.0 (10.5–22.5)
**Rituximab treatment regimens**		
375 mg/m^2^ × 4 weekly infusions (*n*, %)	38 (57.6)	5 (22.7)
1 g 2 weeks apart (*n*, %)	24 (36.4)	13 (59.1)
500 mg 2 weeks apart (*n*, %)	4 (6.1)	3 (13.6)
**Concomitant treatments**		
IV cyclophosphamide (*n*, %)	36 (54.5)	10 (45.4)
IV 6-methylprednisolone (*n*, %)	37 (56.1)	4 (18.2)

*IQR* Interquartile range, *SLEDAI-2 K* Systemic Lupus Erythematosus Disease Activity Index 2000, *BVAS* Birmingham Vasculitis Activity Index, *IV* Intravenous

^a^One patient (indication peripheral polyneuropathy) was excluded from the calculation

**Table 2:** Specific clinical characteristics and pharmacological history of the SLE patients

	**SLE (*n* = 66)**
**Baseline laboratory findings**	
Total leukocytes (*N* cells/mm^3^, median (IQR))	7.0 (4.5–9.1)
Total lymphocytes (*N* cells/mm^3^, median (IQR))	0.9 (0.5–1.4)
CD19 positive cells (*n* = 37) (*N* cells/mm^3^/percentage median (IQR))	0.05 (0.03–0.12)/7.0 (4.0–13.0)
Anti-dsDNA positivity, (*n*, %)	44 (66.7)
Low complement levels (*n* = 61), (*n*, %)	35 (57.4)
**Primary indication for RTX**	
Lupus nephritis, *n* (%)	42 (63.6)
Neurolupus, *n* (%)	7 (10.6)
Lupus related arthritis, *n* (%)	7 (10.6)
Mucocutaneous manifestations, *n* (%)	2 (3.0)
Haematological manifestations, *n* (%)	5 (7.6)
Other manifestations^a^, *n* (%)	3 (4.5)
**Ongoing treatment at RTX initiation**	
Oral Prednisolone, *n* (%)	61 (92.4),
Daily dose, mg (median (IQR))	12.5 (12.5–20.0)
Antimalarials, *n* (%)	27 (40.9)
Azathioprine, *n* (%)	6 (9.1)
Mycophenolate, *n* (%)	9 (13.6)
Methotrexate, *n* (%)	3 (4.5)
**Previous treatments**	
Cyclophosphamide, *n* (%)	40 (60.6)
Cumulative dose (median (IQR))	6075 (3150–9275)
Azathioprine, *n* (%)	44 (66.7)
Mycophenolate, *n* (%)	32 (48.5)
Methotrexate, *n* (%)	17 (25.8)
Cyclosporine A, *n* (%)	11 (16.7)

*IQR* Interquartile range, *n* Number, *CD19* Cluster of differentiation 19, *anti-dsDNA* Anti-double strand-DNA antibodies

^a^Other manifestation: one fatigue and systemic inflammation, one lung fibrosis, one peripheral polyneuropathy

### SLE patients

The majority of SLE patients (Table 1) were females **(**90.9%), with a median (IQR) age of 36.3 (29.8–50.3) years and a disease duration of 7.9 (2.6–14.3) years at the time of RTX initiation. Their disease activity was moderate to high (SLEDAI-2 K median (IQR) 12.0 (7.0–16.0)), and the main indication for RTX use was lupus nephritis (63.6%) (Table 2).

In both disease cohorts, RTX treatment had been administered according to different schedules. The majority of SLE patients (Table 1) were treated according to the haematological regimen (57.6%). Moreover, more than half of the patients had received intravenous cyclophosphamide (54.5%) and 6-metylprednisolone pulse therapy (56.1%) concomitant to RTX. A total of 31 SLE patients were retreated with RTX, 11 of these within one year from their first infusion.

The majority of SLE patients (92.4%) received oral Prednisolone at the time of RTX initiation; however, a majority did not receive concomitant DMARD treatment (72.7%). At baseline, the prevalence of anti-dsDNA positivity was as high as 66.7%, and complement activation was present in 57.4% of SLE patients (Table 2).

### AAV patients

The AAV cohort (*n* = 22) had a median (IQR) age of 62.0 (44.0–70.5) years and a disease duration of 1.5 (0–6.5) years. In this cohort, RTX was primarily administered in a dosage of 1 g 2 weeks apart (59.1%) and a smaller proportion received intravenous cyclophosphamide (45.4%) and 6-metylprednisolone pulses (18.2%) as co-medication (Table 1).

The majority of AAV patients had a diagnosis of granulomatosis with polyangiitis (GPA, *n* = 15) or microscopic polyangiitis (MPA, *n* = 6) accounting for 68.2 and 27.3% of the cohort respectively. Only one patient (4.5%) was included with eosinophilic granulomatosis with polyangiitis (EGPA).

### Frequency of ADAs in SLE patients

A total of three patients (4.8%, 3/62) had detectable ADA at baseline, but all with very low titres (< 2AU/ml). Of these three patients, one progressed at follow-up to develop higher titres of ADA (64 AU/mL) following the first RTX treatment course, one remained positive at very low titre (< 2 AU/mL) and one converted to ADA-negative after treatment.

Follow-up samples from patients with SLE (*n* = 66) were obtained at a median (IQR) of 6.0 (5.0–7.0) months from the first RTX treatment cycle, and immediately prior to the next infusion, if continued treatment was given (five patients were retreated between 6 and 8 months from the first cycle).

ADAs were detected in 37.8% of SLE patients (25/66) at follow-up. Of the positive patients, 6 were positive at low titres (lower or equal to 4 AU/mL), while 19 had higher titres (higher than 5 AU/mL, range 8–4000).

### Frequency of ADA in the AAV patients

At baseline, one AAV patient was detected as ADA-positive but also with a low titre (< 2 AU/ml) and no patient was detected ADA-positive at follow up (*n* = 22), including the patient positive at baseline. The median (IQR) time of follow-up between RTX initiation and follow-up sampling was 5.5 (3.7–7.0) months.

### Association of ADA status with baseline clinical and immunological characteristics

The baseline clinical and immunological characteristics for ADA-positive and ADA-negative SLE patients are shown in Table 3.

**Table 3:** Comparison of the baseline characteristics of ADA-positive and ADA-negative SLE patients

	**ADA positive (*n* = 25)**	**ADA negative (*n* = 41)**	*P* value*
**Patient characteristics**			
Age (years, median (IQR))	34.0 (25.9–40.8)	44.3 (32.7–56.3)	***0.002***
Sex (Females, *n*, %)	27 (87.1)	33 (94.2)	0.66
Disease duration (years, median (IQR))	4.14 (1.18–10.08)	9.19 (5.71–16.93)	***0.0097***
**Disease activity**			
SLEDAI-2 K (median (IQR))	14.0 (10.0–18.5)	8.0 (6.0–14.0)	***0.017***
**Laboratory data**			
Total leukocytes (*N* cells/mm^3^ median (IQR))	6.3 (4.1–8.1)	7.1 (4.7–9.5)	0.53
Total lymphocytes (*N* cells/mm^3^ median (IQR))	0.98 (0.54–1.47)	0.85 (0.5–1.52)	0.74
CD19 + cells (%, median (IQR))	8.0 (5.25–14.5)	6.0 (2.5–11.5)	0.17
CD19 + cells (*N* × 10^9/L, median (IQR))	0.065 (0.03–0.15)	0.05 (0.03–0.085)	0.21
**Treatments**			
Concomitant CS pulses, % (*n)*	60.0 (15)	53.6 (22)	0.61
Concomitant cyclophosphamide, % (*n)*	56.0 (14)	53.6 (22)	0.85
Cumulative dose of concomitant cyclophosphamide (mg, (median (IQR))	1200 (950–1600)	1000 (1000–1600)	0.81
Previous cyclophosphamide, % (*n)*	60.0 (15)	60.0 (25)	0.93
Cumulative dose of previous cyclophosphamide (mg, median (IQR))	6700 (4500–10000)	6000 (3000–9150)	0.58
Corticosteroids at baseline, % (*n*)	92 (23)	92.6 (38)	0.91
Total corticosteroid dose at baseline (mg, median (IQR))	12.5 (10–17.5)	15.0 (7.5–20.0)	0.73
Antimalarial treatment at baseline, % (*n)*	36.0 (9)	43.9 (18)	0.53
DMARDs at baseline (AZA, MMF, MTX), *n*	0/4/1	6/5/2	n/a
**Rituximab treatment (from baseline)**			
Schedule of RTX (*n*, 375 mg/m^2^ × 4, 1 g × 2, 500 mg × 2))	14/10/1	24/14/3	1.00
Cumulative dose of RTX at first course (mg, median (IQR))	2100 (2000–2650)	2400 (2000–2800)	0.33

*IQR* Interquartile range, *SLEDAI-2 K* SLE Disease Activity Index-2000, *CD19* Cluster of differentiation 19, *RTX* Rituximab, *n/a* Not applicable, *DMARDs* Disease-modifying anti-rheumatic drugs, *AZA* Azathioprine, *MMF* Mycophenolate mofetil, *MTX* Methotrexate, *CS* Corticosteroids

^*^Mann–Whitney test

**Fig. 1:**
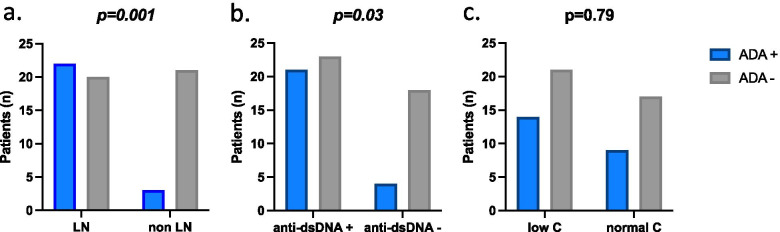
Association of ADA status at follow-up with baseline clinical indication for treatment and immunological features. **a** Frequency of ADA positivity at follow-up is significantly higher in SLE patients treated for lupus nephritis (LN) with respect to other clinical manifestations (non-LN), Fisher exact test, *p* = 0.001. **b** ADA positivity at follow-up is associated with the presence of positive anti-dsDNA at baseline, Fisher exact text, *p* = 0.03*.***c** No significant association of complement (C) activation at baseline with positive ADA status at follow-up (chi-square test)

In the univariate analysis (Table 3), ADA-positive SLE patients were significantly younger compared to ADA-negative (median (IQR) 34.0 (25.9–40.8) vs 44.3 (32.7–56.3) years, *p* = 0.002) and with shorter disease duration (median (IQR) 4.14 (1.18–10.08) vs 9.19 (5.71–16.93, *p* = 0.0097). ADA-positive patients also had a higher baseline disease activity (median (IQR) SLEDAI-2 K 14.0 (10.0–18.5) vs. 9.19 (5.71–16.93), *p* = 0.0017). Patients being treated for lupus nephritis had a higher rate of ADA compared to SLE patients treated for other indications (22/42, (52.4%) vs 3/24, (15.5%), Fisher exact test, *p* = 0.001, Fig. 1a).

Baseline anti-dsDNA positivity was also significantly associated with ADA positivity at follow-up with 47.7% (21/44) of anti-dsDNA-positive patients baseline developing ADA compared to 18.2% (4/22) of the ADA-negative patients (Fisher’s exact test *p* = 0.03, Fig. 1b). Conversely, the anti-dsDNA positive patients were also more prevalently affected by lupus nephritis (*n* = 32, 72.7%).

No difference was seen in ADA development between groups regarding RTX treatment schedule (*p* = 1.00) or cumulative dose (*p* = 0.33). Furthermore, no difference was seen between ADA-positive and negative groups in previous exposure and concomitant treatment with intravenous cyclophosphamide (chi-square test, *p* = 0.93 and *p* = 0.58, respectively) or intravenous corticosteroid pulse therapy at time of RTX infusion (chi-square test, *p* = 0.91). Similarly, the use of antimalarials, oral corticosteroids or DMARDs prior to RTX was not associated with the presence of ADAs at follow-up (Table 3). Finally, no association was seen between ADA and baseline complement activation (chi-squared test, *p* = 0.79, Fig. 1c).

### Association of ADA development and peripheral B cell depletion

Data on peripheral blood lymphocytes subpopulations as measured by flow cytometry were available for 37 of the SLE patients at baseline and 37 patients at 6 months of follow-up.

No statistically significant differences were found regarding the baseline B cell count between ADA-positive and negative SLE patients at follow-up.

**Fig. 2:**
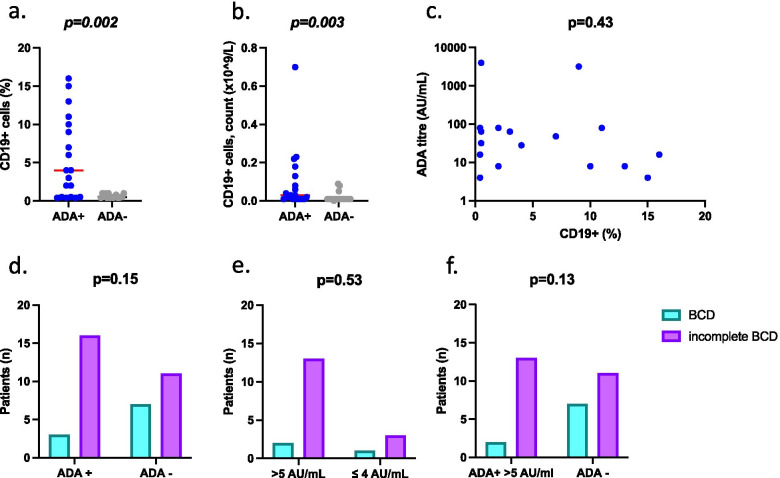
Pharmacodynamic differences on B cells between ADA + and ADA- patients at 6 months follow-up. **a** Percentage CD19 + cells in the peripheral blood 6 months after rituximab infusion, showing significantly higher values in all ADA positive patients compared to ADA negative patients (*p* = 0.002). Red line shows the group median. **b** ADA-positive patients have a significantly higher median value of CD19 + cells in the peripheral blood expressed as absolute numbers per microliter (*p* = 0.003). Red line shows group median. **c** Correlation (Spearman rho) between the titers of ADA (expressed as AU/mL) and the peripheral B cell count at 6 months follow up (expressed as CD19%) did not show any correlation (*p* = 0.43, *r* =  − 0.2 95%CI − 0.63–0.32). **d** At 6 months after rituximab infusion, no statistically different rate of achievement of B cell depletion was seen between ADA-positive and ADA-negative groups; B cell depletion (BCD) defined as peripheral blood CD19% < 0.5. **e** At 6 months after rituximab infusion, ADA positive were stratified into either higher ADA titre (above 5 AU/mL) and lower ADA titre (lower or equal to 4 AU/mL) and show no statistically different rate of achievement of BCD. **f** No difference was observed when comparing rates of BCD between ADA negative and high ADA positive (above 5 AU/mL)

At 6 months follow-up, a higher proportion of CD19 + to total lymphocytes was seen in ADA-positive patients compared to ADA-negative patients (median (IQR) 4.0 (0.5–10.0) vs 0.5 (0.4–1.0), *p* = 0.002) (Fig. 2a). Similarly, when comparing the absolute count of CD19 + cells in the peripheral blood, significant differences were observed, with a median (IQR) 0.03 (0.01–0.14) vs 0.01 (0.01–0.01), *p* = 0.003) comparing ADA-positive and ADA-negative patient groups (Fig. 2b). A higher proportion of patients with ADA-positive status at follow-up did not achieve the status of B cell depletion (CD19% in peripheral blood < 0.5%), although this did not reach statistical significance (Fig. 2d-f).

No significant correlation was found between the ADA status and the proportion of patients who achieved B cell depletion (BCD) at follow-up (Fig. 2d). Thereafter, we stratified ADA-positive patients by low titre (lower or equal to 4 AU/ml), and high titres (higher than 5 AU/ml).

No significant difference was seen between B cell depletion in low and high ADA-positive titre groups (Fig. 2e). Moreover, when comparing higher titre ADA-positive patients to negative ADA patients, it was not statistically significant (Fig. 2f).

### Adverse events associated with ADA at retreatment

Thirty-one of the 66 SLE patients underwent retreatment over time. Of these, 12 (38.7%) were ADA-positive and retreated at a median of 17.5 (10.2–66.7) months after the first infusion. The remaining 19 patients (61.3%), who were ADA-negative, were retreated after a median of 19.0 (9.7–67.0) months following the first RTX infusion.

Of the 12 ADA-positive SLE patients who were retreated, three (25.0%, 3/12) had immediate infusion reactions and two (16.7%) manifested serum sickness following the second course of RTX treatment. Of the three patients with immediate infusion reactions two had an ADA titre of 80 AU/mL and one a titre of 8 AU/mL and were retreated at 10, 11 and 115 months from the first course of RTX respectively. The two SLE patients who developed serum sickness had an ADA titre of 4000 AU/mL and 4 AU/mL respectively and retreatment was administered at 8 and 23 months respectively from the first course of RTX. There were no immediate infusion reactions in ADA-negative patients at retreatment however, one patient presented with a late-onset reaction suggestive of serum-sickness (5.2%, 1/19).

### Variations in disease activity (SLEDAI-2 K) after rituximab treatment

Overall, the entire group of SLE patients responded with a reduction of median SLEDAI-2 K of eight units, from 12.0 (7.0–16.0) at baseline to 4.0 (2.0–6.7) at 6 months follow-up (Wilcoxon signed-rank test *p* < 0.0001). A significant reduction of disease activity was observed both in ADA-positive and ADA-negative patients (*p* < 0.0001 in each subgroup*)*. However, the ADA-positive patients still had a higher disease activity at 6 months follow-up compared to ADA-negative patients (Mann–Whitney test *p* = 0.004). Stratifying by indication to RTX initiation, a reduction of disease activity was evident both for SLE patients treated for lupus nephritis and for those treated for all other indications (Wilcoxon signed-rank test *p* < 0.0001 for both subgroups).

**Fig. 3:**
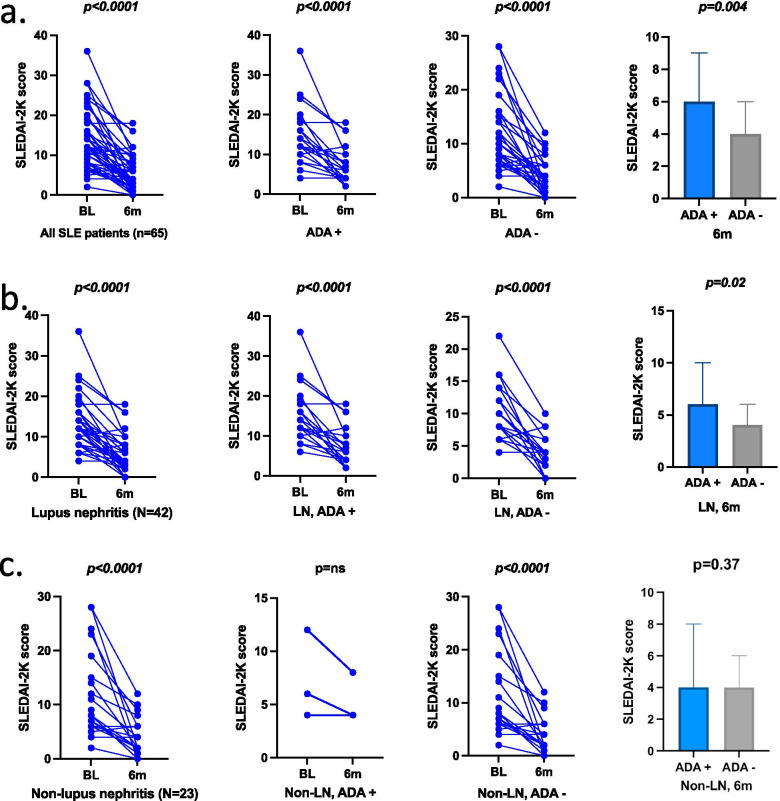
Changes in disease activity in SLE patients, as measured by SLEDAI-2 K. **a** The SLEDAI-2 K score shows a significant reduction in the whole SLE cohort (*N* = 65, one value missing) from baseline to 6-months follow-up, with significant reduction that is confirmed also for patients ADA-positive and ADA-negative at 6-months follow-up (Wilcoxon signed-rank test, *p* < 0.0001). When evaluated at 6-months follow-up, the ADA-positive patients showed however higher SLEDAI-2 K values compared to ADA-negative patients (Mann–Whitney test *p* = 0.004). **b** Similar behaviour, with significant reduction of SLEDAI-2 K between baseline and 6-months follow-up, was observed for lupus nephritis (LN) patients and maintained whether they were ADA-positive or ADA negative at follow-up (Wilcoxon signed-rank test *p* < 0.0001 for all)*.* Also in LN patients, SLEDAI-2 K values at 6-months follow-up were higher in ADA-positive patients compared to ADA-negative ones (Mann–Whitney test, *p* = 0.02). **c** Patients treated for clinical indications other than lupus nephritis (non-lupus nephritis, non-LN) also showed a significant reduction of the SLEDAI-2 K at a group level and in both ADA-positive and ADA-negative subgroups (Wilcoxon signed-rank test *p* < 0.0001, *p* = ns and *p* < 0.0001, respectively). In these patients, SLEDAI-2 K values, measured at 6-months, did not differ in a statistically meaningful manner, between ADA-positive and ADA-negative subgroups (Mann–Whitney test, *p* = 0.37)

In the lupus nephritis group, the patients ADA-positive at follow-up, showed a significant reduction of the SLEDAI-2 K score from baseline to 6 months follow-up (*p* < 0.0001), similarly to ADA-negative lupus nephritis patients (*p* < 0.0001). However, their disease activity was higher at follow-up compared to the ADA-negative patients with lupus nephritis (*p* = 0.02) (Fig. 3).

### Efficacy of RTX after retreatment in lupus nephritis ADA-positive patients

Among the 42 SLE patients who had been started on RTX for active lupus nephritis, a total of 17 had undergone retreatment at some point (median (IQR) time to retreatment 38.0 (11.5–81.0) months). Of these 17 patients, 9 were found to be ADA-positive 6 months after the first RTX course (before retreatment), with a median (IQR) ADA titre of 48.0 (6.0–80.0) AU/mL. Seven of these retreated lupus nephritis patients (7/17) had early retreatment, i.e. within 18 months from the nephritis flare for which RTX was started, two for non-nephritic flares (one ADA-positive and one ADA-negative) and five for nephritic flares (four of which ADA-positive), respectively.

Of the latter four ADA-positive patients who had a nephritic flare requiring early retreatment, three were non-responders and one partial responder at 6 and 12 months from retreatment [25]. All except for one patient with ADA titre < 2 AU/mL, were found to have high titres of ADA (48, 64 and 4000 AU/ml, respectively).

## Discussion

In this study, we showed that RTX treatment is associated with a high rate of ADA positivity in SLE patients already after the first course of treatment. Moreover, ADA occurrence was not influenced by concomitant administration of intravenous corticosteroids or immunosuppressants such as cyclophosphamide. Among the SLE patients, ADAs were mainly observed in patients with nephritis and a serologically active profile with anti-dsDNA antibody positivity. In contrast, the development of ADAs was not observed in AAV patients after exposure to the first course of RTX.

The development of ADAs (in the previous literature also described as HACA, human-anti-chimeric antibodies) has been reported in early clinical trials [4] evaluating RTX in SLE. In this context, ADAs were more commonly observed in patients who exhibited higher disease activity at baseline and were of Afro-American ethnic background. In addition, when in high titres, they were associated to less efficient B cell depletion and lower RTX serum concentrations. Similarly, the two RCT [6, 7] evaluating RTX in SLE report on the presence of ADA (HACA), in 26.1% and 15.1% of the patients in the active treatment arm, with some patients experiencing adverse events. More recently, studies on real-life cohorts, have underscored how ADA can be implicated in the loss of clinical efficacy and less efficient B cell depletion of peripheral blood at retreatment in SLE patients [14].

In our study, we reported a frequency of 37.8% ADA positivity in SLE patients after the first exposure to RTX, consistent with the 37% reported by Wincup et al., who also used a bridging ECL immunoassay for ADA detection. Similarly, in line with their findings, our ADA-positive patients were younger at the first exposure to RTX compared to ADA-negative individuals [15]. This is in contrast with observations concerning ADAs against other biotherapies, such as interferon-beta in MS, where ADA-positive patients were significantly older than patients with persistently negative results for ADA testing [28].

Disease activity at baseline, was higher in ADA positive patients in our study, which contrasts to what was described by Wincup et al. [15], probably reflecting the fact that our cohort was more skewed towards a higher prevalence of severe lupus manifestations including active nephritis and CNS patients. This could also explain how baseline immunological features such as anti-dsDNA positivity were found to be associated to ADA positivity at follow-up in our study. Even though such features may differentiate our patients from other cohorts, it is relevant to highlight how the patients more prone to develop ADA in our SLE cohort, were those with refractory organ involvement such as lupus nephritis and positivity for anti-dsDNA. This observation is of major relevance, since it underlines how patients more in need of additional treatment, who are the usual candidates to RTX, are also those more susceptible to ADAs development. Also, this highlights that the immunogenicity of RTX may be strongly associated to patient-related factors, in this case, the immunological and inflammatory status of the patients. Comparably, the major determinant of the development of ADAs to interferon-beta in MS was found to be high disease activity during treatment exposure [28].

As suggested by previous studies, systemic diseases seem to be more prone than RA in developing immunogenicity to RTX. In a retrospective analysis by Combier et al., having a systemic disease was a major factor associated to the presence of ADAs. The study though included only fifteen SLE patients, and five AAV, as the majority of systemic illnesses were represented by Sjögren’s syndrome [12].

We observed that peripheral blood B cell counts at follow-up were higher in ADA-positive patients compared to ADA-negative, which contrast with a recent report [15] but is consistent with an aforementioned clinical trial [4]. However, if these higher B cell counts reflect a less efficient B cell depletion or an accelerated repopulation, remains unclear.

Although the B cell counts could be influenced by diverse modalities of data collection and power of the studies, this might suggest that ADAs could influence the pharmacokinetic and pharmacodynamic of the drug leading to pharmacologic abrogation and reduced therapeutic exposure. Despite enrolling a higher number of patients compared to previous studies, the reduced availability of data concerning the peripheral B cell counts resulted in a lack of power for our study. Therefore, we were not fully able to evaluate the association between ADA titres and B cell counts or depletion as a surrogate of RTX efficacy.

In fact, we could not observe a strong and direct correlation between ADA titres and B cell counts. This may also be explained on a pharmacological level. ADAs above a certain threshold, which exceeds the amount of circulating drug, may saturate the available drug and form immune-complexes but not impact further on the reduction of B cell count which follows RTX action.

Moreover, although we observed a difference in peripheral B cell counts between ADA-positive and ADA-negative patients, we could not confirm this difference when we further examined the rate of achievement of B cell depletion. Furthermore, it is important to note that, given we evaluated patients in this study after the first exposure to RTX, the interpretation of the effect of ADAs on B cell count following immunization to the drug is incomplete. Indeed, the real impact on B cell depletion efficiency might be expected upon retreatment.

In line with previous observations, we could confirm that the immunogenicity of RTX was not influenced by the concomitant administration of immunosuppressants such as intravenous corticosteroids and cyclophosphamide. This contrasts to other autoimmune diseases such as RA, where the concomitant treatment with methotrexate has been shown to be protective against, or at least delaying, the development of ADAs against infliximab, another chimeric monoclonal antibody [29].

Of interest is the striking difference between the ADA frequencies in SLE and AAV, as these might reflect different disease associated ADA risk factors in terms of RTX immunogenicity. On an immunological level, although both autoimmune in nature, the two diseases are characterized by different levels and quality of B cell reactivity and the propensity to respond to biotherapeutics with ADA formation in SLE compared to AAV, might be related to a more profound disruption of B cell tolerance [30, 31].

This difference might also be implicated in the fact that we also found that a small portion of SLE samples, although naïve to RTX, were ADA-positive at low titre (< 2 AU/mL), which although a rare event was still higher than in AAV. These ADAs were still above the disease-specific cut-point established according to internationally established recommendations [32] and suggest the presence of naturally occurring antibodies, which in turn, should reflect the intrinsic B cell hyperreactivity typical of SLE and the presence of a remarkably broad antibody repertoire.

With respect to safety, our retrospective analysis of the clinical records allowed us to trace immediate and late-onset reactions in the ADA-positive patients who were retreated with RTX.

However, while the recording of immediate reactions was performed directly at the hospital, those of the late-onset reactions mostly relied on patients reporting such reactions afterwards.

Therefore, the possibility arises that our study might underestimate the true occurrence of serum sickness reactions.

We could also observe that ADA-positive patients who received a second course of RTX for early nephritic flares, did not respond in general, and all had higher ADA titres, though the low numbers in our study do not allow to make generalizations.

Taken together, our findings suggest the necessity of an increased awareness for the immunogenic consequences of RTX administration in SLE, while such phenomenon appears not to be of greater relevance for AAV. Based on our findings, we believe screening for ADA should be implemented following RTX treatment, in particular in younger patients with lupus nephritis as a leading manifestation. In such patients, retreatment should be considered carefully, both for possible lack of efficacy and for safety reasons, and alternative B cell depletive drugs could be considered in the case of ADA positivity. Further investigations are also needed to evaluate the rate of immunogenicity of RTX biosimilars in the context of SLE.

Further research should also address the question on whether RTX doses currently used might play a role in determining the immunogenicity of the drug and whether repeated treatments at regular intervals may affect and mitigate it. Indeed, in our cohort, given the large timespan to retreatment, possible effects of repeated exposure to RTX in terms of tolerization could not be investigated. A deeper comprehension of the immunological and clinical impact of ADA in RTX treated SLE patients warrants the conduction of longitudinal studies in order to better define which patients should stop RTX and/or be switched to alternative anti-CD20 agents.

## Conclusions

To conclude, our study presents the result of a large real-life cohort of SLE patients in which we could demonstrate a high occurrence of ADA to RTX after the first exposure, highlighting how the patients more in need of RTX are also the ones more affected by the phenomenon of immunogenicity. In comparison to AAV, the reason for immunogenicity may be related to intrinsic immunological features of SLE. We believe that our findings should encourage further studies and the implementation of screening of SLE patients in the clinical routine.

## Data Availability

Data collection has been conducted in accordance to local regulation. In the original dataset, data are registered anonymously. The authors declare their availability in providing data if requested by the referees or the editorial team of the journal.

## Abbreviations

ADAs/ADA: Anti-drug antibodies
RTX: Rituximab
SLE: Systemic lupus erythematosus
AAV: ANCA-associated vasculitis
ANCA: Anti-neutrophil cytoplasmic antibodies
GPA: Granulomatosis with polyangiitis
MPA: Microscopic polyangiitis
EGPA: Eosinophilic granulomatosis with polyangiitis
RA: Rheumatoid arthritis
MS: Multiple sclerosis
SLEDAI-2 K: Systemic Lupus Erythematosus Disease Activity Index-2000
BVAS: Birmingham Vasculitis Activity Score
ACR: American College of Rheumatology
EMA: European Medicine Agency
NIH: National Institutes of Health
DMARDs: Disease-modifying anti-rheumatic drugs
AZA: Azathioprine
MMF: Mycophenolate mofetil
MTX: Methotrexate
CS: Corticosteroids
IR: Infusion reactions
ECL: Electrochemiluminescence (immunoassay)
MSD: Meso Scale Discovery (platform)
AU/mL: Arbitrary unit per millilitre
Anti-dsDNA: Anti-double strand-DNA antibodies
U-ACR: Urine-albumin to creatinine ratio
BCD: B cell depletion
CD19: Cluster of differentiation-19
CD20: Cluster of differentiation-20
HACA: Human anti-chimeric antibodies
IQR: Interquartile range
CNS: Central nervous system
*N*: Number
